# Progress in Psychiatry

**Published:** 1901-11-30

**Authors:** 


### PROGRESS IN PSYCHIATRY.

(*Continued from page* 67.)

**Acute Febrile Delirium with Hallucinations.**—This group of mental disorders includes some cases of puerperal insanity, acute delirium proper (so-called acute delirious mania), and the febrile delirium associated with influenza, scarlet fever, acute cerebro-spinal meningitis, tubercular meningitis and allied infections. Acute delirium in the strict sense is an acute phrenopathy or insanity characterised by intense delirium, pyrexia, total incoherence and marked bodily excitement, and rapid exhaustion tending in the majority of cases to death. Owing to the profound exhaustion it produces combined with excitement it has been designated typho-mania (*i.e.*, mania *plus* a typhoid or adynamic condition) by Luther Bell who first described it in 1849. Authorities differ as to whether it is a definite clinical entity. In some instances general paralytics in the course of their disease develop symptoms of acute delirium and exhaustion, the cause of which is obscure, but which probably depend upon some infection or toxic agency to which such debilitated patients are prone. For such cases the term "fulminating form of general paralysis" has been applied, a designation which has the merit of elasticity but explains nothing. The condition of cerebral and bodily debility which may exist in puerperal women or in alcoholic subjects is also one which favours the development of acute delirium ; in fact this disease may develop on a basis of cerebral and bodily exhaustion however caused—*e.g.*, worry, starving, trauma, cancer and the like. Thus instances of its occurrence have been recorded after a host of infectious disorders such as pneumonia, typhoid fever, influenza, acute rheumatism, puerperal fever, dysentery (Soukhanoff), phthisis (Holst [17]) and other affections.

The disease is, however, of rare occurrence, and it is probable from the results of bacteriological examination of the blood of patients suffering from it (Ceni, Bianchi, and others) that streptococci, staphylococci, and a bacillus described by Bianchi [18] as being present in the blood, play a part in the ætiology of the disease. In a considerable proportion of cases acute delirium may arise in the course of chronic insanity, in which case spmptoms of exhaustion and paralysis of functions are very marked, and a fatal termination is the usual result. As regards age it has been found that the majority of cases occur between the ages of 25 and 45 years. Thus in a series of 33 cases, recorded in 1895 by Christensen, a Danish physician, only one was below 25 ; 21 patients were between 25 and 45 years ; 9 were between 45 and 65 ; and the remaining 2 were above 65. Of 45 examples collected by Krafft-Ebing all were between the ages of 27 and 47 years. The proportion of the sexes was almost exactly equal in Krafft-Ebing's cases, but according to Christensen, Spitzka, and others, female cases preponderate more or less. The clinical symptoms are essentially those of collapse or exhaustion, rapidly tending to a typhoid condition, pyrexia, complete incoherence, twitchings, and restlessness, or great excitement and intractable insomnia. The onset is marked by insomnia, mental apathy and confusion, together with headache, lassitude, and incapacity for thought or action. Then follow symptoms of fever, hallucinations of the senses, increasing general bodily exhaustion, loss of appetite and of flesh. The headache increases in severity, the patient is restless and tumbles or stumbles about in a condition of sub-acute delirium, and apparently in a state of automatism and mental confusion ; he staggers about, the flesh is very prone to develop bruises, the skin is hot and dry, and the breath offensive. The pulse is rapid, and usually about 120 per second. Constipation, and later offensive diarrhœa are present. The symptoms of excitement are unlike those of simple mania, and are more closely allied to stupor and hallucinatory delirium, and sometimes suggest those of cerebral meningitis. Both *tache cerebrale* and excessive irritability and response of the muscles to local percussion (myoidema) are frequently present. The reflexes are exaggerated and convulsive reactions readily provoked. Facial twitchings and oculopupillary disturbances may be present, and give additional resemblance to symptoms of meningitis. The temperature is four or five degrees above the normal. The next stage is characterised by an acute delirium in the stricter sense. A wild phrenzy with restlessness and excitement follows, and it is seen that the patient is suffering from delusions or hallucinations of terror. Owing to the extreme degree of unrest it is difficult to feed the patient by the tube or by means of enemata. Food given by the mouth is often spat out. The body wastes rapidly, and profound exhaustion now set in. Spitzka has described symmetrical atrophy of certain groups of muscles in acute delirium. A profound trophic and vaso-motor paresis shows itself at this stage, and may be characterised by the appearance of pemphigus or bullæ on the extremities, and by the occurrence of bed-sores, and even gangrene. Evacuations are now passed unconsciously, and death occurs suddenly from syncope or gradually from coma and exhaustion. The prognosis is grave in all cases. "The majority of the patients die after an illness of a few weeks. Complete recovery never occurs, the patients being crippled both mentally and physically during the rest of their lives" (Macpherson [19]). The pathology of acute delirium is a mixed one. The disease never occurs *de novo* in healthy subjects, but is only met with in those who have been debilitated by various agencies—labour and the puerperium, septicæmia, alcoholism, cerebral traumatism, insolation, and pneumonia or other fevers. Patients who have been the subjects of one or more attacks of insanity and are tending toward chronicity, and neuropathic subjects generally, may contract this disease. On this basis of cerebral exhaustion or debility, acute delirium may develop. The clinical symptoms indicate that some acute infective process —analogous to acute cerebro-spinal meningitis—is the superadded agency. Careful bacteriological examinations have been made by various observers towards establishing such a conclusion. Thus Rasori (1894) found and described a bacillus present in a softened area of the brain in a fatal case of acute delirium. This organism had rounded ends, grew in ordinary nutrient media, and was stained by the ordinary aniline dyes, but not by Grain's method. Rabbits inoculated beneath the dura mater with pure cultures of this bacillus died in two days, and in cases of hypodermic inoculation they died in four to six days. In both forms of inoculation ending fatally, post-mortem examination showed the presence of cerebral congestion (hyperæmia) and œdema. Sir John Batty Tuke [20] (1897) also found a bacillus in the urine and blood of a fatal case of the disease, and previous observers had shown that the bacillus and diplococcus of croupous pneumonia, and the streptococcus pyogenes aureus were frequently found in the blood or in the cerebro-spinal exudates. Bianchi (1893) and other Italian observers have also found a bacillus and streptococci (including diplococci) in some cases, and the colon bacillus has been found in others. The consensus of bacteriological evidence thus shows that one or more of the following organisms may be present—streptococcus pyogenes aureus, diplococcus pneumoniæ et meningitidis, pneumobaccillus, special bacilli of Rasori, Tuke, and Bianchi, and bacterium coli communis. The pathological changes met with in the brain and nervous system generally indicate the presence of an acute infective process. Intense hyperæmia, with punctiform hæmorrhages are present in the cerebral cortex. The cerebral lesions include extreme chromatolysis and granular degeneration of the cortical nerve-cell bodies as shown by the aniline and Nissl methods, profound degenerative changes in their protoplasmic processes (destruction of "gemmules" and conspicuous atrophy with varicosities of the dendrites), and varying degrees of degeneration in the medullated fibres (as shown by Marchi's and

Weigert's methods) in all layers of the cerebral and cerebellar cortex. Similar changes are found in the bulbo-spinal nuclei and white substance, but the maximum degree of lesion presents itself in the cerebral cortex. The neuroglia cells in the affected regions show moderate degrees of hypertrophy and hyperplasia, and the connective tissue cells in the perivascular sheaths and pericellur sacs of the brain exhibit a similar overgrowth. Some degree of diapedesis of white corpuscles is present in the meninges along the vessels. There are in fact many points of resemblance in the pathological changes between fatal cases of puerperal delirium, subacute alcoholism, general paralysis running a very acute course, and acute delirium. It is possible that there are two or three varieties of acute delirium, viz., a group with cocci in the blood (streptococci, diplococci, and staphylococci), a bacillary group (bacillus of Bianchi, Rasori, and others, the colon bacillus and the pneumobacillus), and a mixed group (with mixed infection). The treatment of acute delirium includes careful, frequent, and generous feeding with liquid and easily assimilable diet which is the best stimulant and hypnotic combined, cleanliness of the body and bedding and the use of aseptic lotions, so as to ward off bed-sores, the cleansing of the patients' mouth, lips and teeth with aseptic wet lint or swabs, aperients in the early stage so as to open the bowels freely and thus lessen auto-intoxication from the bowel, and restraint in bed with sheets or with a strait shirt so as to prevent excessive restlessness and exhaustion. The patient should be kept in a quiet and darkened room, and all excitement avoided. Chloral is not good as it increases the cerebral venous congestion and chills the body ; sulphonal and sodium bromides are safer hypnotics though little effective as a rule ; digitalis should be given in moderate or large doses when heart failure is threatened, and, according to Kellog, ergotin is an excellent drug to diminish the excitement. On the analogy of puerperal septic cases threatening a fatal issue and which have occasionally reacted well to venesection and normal saline transfusion, this may be tried almost as a last resource.

[17] Neurol. Centr., 1885. [18] Annali di Neurologia, 1893, 1895 and 1899. [19] Mental Affections, 1899. [20] Edinb. Med. Jour., 1897

